# Neurofibromin haploinsufficiency results in altered spermatogenesis in a mouse model of neurofibromatosis type 1

**DOI:** 10.1371/journal.pone.0208835

**Published:** 2018-12-20

**Authors:** Harleen Chohan, Mitra Esfandiarei, Darian Arman, Catherine D. Van Raamsdonk, Cornelis van Breemen, Jan M. Friedman, Kimberly A. Jett

**Affiliations:** 1 Department of Medical Genetics, BC Children Hospital Research Institute, University of British Columbia, Vancouver, Canada; 2 Department of Anesthesiology, Pharmacology and Therapeutics, BC Children Hospital Research Institute, University of British Columbia, Vancouver, Canada; 3 Department of Biomedical Sciences, College of Graduate Studies, Midwestern University, Glendale, Arizona, United States of America; University Hospital of Münster, GERMANY

## Abstract

The fertility of men with neurofibromatosis 1 (NF1) is reduced. Despite this observation, gonadal function has not been examined in patients with NF1. In order to assess the role of reduced neurofibromin in the testes, we examined testicular morphology and function in an *Nf1*^*+/-*^ mouse model. We found that although *Nf1*^*+/-*^ male mice are able to reproduce, they have significantly fewer pups per litter than *Nf1*^*+/+*^ control males. Reduced fertility in *Nf1*^*+/-*^ male mice is associated with disorganization of the seminiferous epithelium, with exfoliation of germ cells and immature spermatids into the tubule lumen. Morphometric analysis shows that these alterations are associated with decreased Leydig cell numbers and increased spermatid cell numbers. We hypothesized that hyper-activation of Ras in *Nf1*^*+/-*^ males affects ectoplasmic specialization, a Sertoli-spermatid adherens junction involved in spermiation. Consistent with this idea, we found increased expression of phosphorylated ERK, a downstream effector of Ras that has been shown to alter ectoplasmic specialization, in *Nf1*^*+/-*^ males in comparison to control *Nf1*^*+/+*^ littermates. These data demonstrate that neurofibromin haploinsufficiency impairs spermatogenesis and fertility in a mouse model of NF1.

## Introduction

Neurofibromatosis 1 (NF1) is a multisystem autosomal dominant condition that affects 1 in 3000 individuals. It is caused by germ-line mutations in the neurofibromin (*NF1*) gene, which is located on chromosome 17. There is significant variation in the clinical phenotype among affected individuals, but the most common manifestations are café-au-lait macules, iris Lisch nodules, multiple neurofibromas, axillary and inguinal freckling, skeletal abnormalities and learning disabilities [1]. Individuals with NF1 have fewer children, on average, than individuals in the general population, and this reduction in fertility is greater in males than females [2–6]. This reduction in reproductive fitness has been attributed to social rather than biological factors [2, 4], but gonadal function has rarely been examined in patients with NF1. One, German study [7] found decreased libido in males with NF1 and one French study [8] described testicular dystrophy.

Recent studies demonstrate that the reproductive period of individuals with NF1 is often reduced suggesting that gonadal function may be impaired. Children with NF1 have been found to enter puberty later than their unaffected siblings or children in the general population [9]. The onset of menstruation in girls with NF1 is delayed when compared to their unaffected mothers or unaffected girls in the general population [10]. Women with NF1 also often enter menopause earlier than expected [11]. In addition, a higher than expected occurrence of osteoporosis has been reported in men with NF1 [11], which may reflect alterations in the production of testicular hormones [12].

Spermatogenesis, the process by which spermatogonia become haploid spermatids, takes place in the seminiferous epithelium and is dependent on multiple cell-cell interactions [13, 14]. The seminiferous epithelium is composed of spermatogonia, spermatocytes, spermatids, mature spermatozoa and Sertoli cells. Spermatogonial stem cells lie on the basal lamina where they proliferate and differentiate into spermatocytes. Following two meiotic divisions, spermatocytes become round spermatids that eventually differentiate into elongated spermatids and then mature spermatozoa. Sertoli cells help regulate this process through the production of growth factors and cytokines. Interstitial Leydig cells and the hypothalamic-pituitary axis provide additional support for the process through the production of hormones [15–17]. Neurofibromin, the protein product of the *NF1* gene, is highly expressed in developing and adult gonads [18], where its expression occurs during spermatogenesis and the estrus cycle [18]. Neurofibromin is a Ras GTPase-activating protein involved in regulation of the Ras/mitogen activated protein kinase (MAPK) signaling pathway that controls cell division [19]. Ras signaling is essential for proper spermatogenesis and is also important for the proliferation and migration of germ cells and maintaining the blood-testis-barrier [20–25].

Considering the importance of Ras signaling for the proliferation of spermatogonia and for the maintenance of junctional dynamics within Sertoli cells and spermatids [22, 26, 27], and its dysregulation in NF1 [19, 28], we hypothesized that haploinsufficiency of neurofibromin has negative effects on spermatogenesis in males with NF1. We tested this hypothesis by studying the effects of neurofibromin deficiency on cellular differentiation and migration within the seminiferous epithelium in a well-established mouse model of NF1, the *Nf1*^Dsk9/+^ mouse.

## Materials & methods

### Experimental animals

The NF1 mouse model used in this study has been described elsewhere [29]. *Dsk9* is a missense mutation in the GTPase-activating protein related domain of neurofibromin. *Nf1*^Dsk9/+^ (subsequently referred to as “*Nf1*^+/-^”) mice were maintained on a mixed (C3HeB/FeJ x C57bl/6) background. All surgical and animal care procedures were approved by the University of British Columbia Committee on Ethics of Animal Experiments (IACUC Approval # A11-0117), and were conducted in accordance to the University’s Guidelines for Animal Experiments (https://animalcare.ubc.ca/animal-care-committee/policies-and-guidelines). For the breeding studies, male or female *Nf1*^+/-^ mice were bred with *Nf1*^*+/+*^ control littermates. For breeding studies, we used three haremed cages in which female *Nf1*^+/-^ mice were crossed to male *Nf1*^*+/+*^ control littermates and three haremed cages in which male *Nf1*^+/-^ mice were crossed to female *Nf1*^*+/+*^ control littermate. We also examined breeding records from additional six cages using the same crosses that were not haremed.

### Tissue isolation procedure

*Nf1*^+/-^ and *Nf1*^*+/+*^control littermate mice were weighed prior to surgical procedures. Animals were sacrificed using isoflurane followed by cervical dislocation at 6 to 7 months of age. The testes and epididymis were isolated from the lower abdominal cavity and placed in ice-cold HEPES-PSS buffer (PH 7.4) containing 10mM HEPES, 6mM glucose, 1.8mM CaCl, 130mM NaCl, 4mM KCl, 4mM NaHCO_3_, 1.2mM MgSO_4_, 1.2mM KH_2_PO_4_, and 0.03mM EDTA. The testes were cleaned of fat, weighed, and either fixed in 10% formalin or flash-frozen in liquid nitrogen and stored at -80°C for histological staining and Western blotting, respectively. Within 30 minutes of isolation, the cauda epididymis, where mature sperm are stored, was cleaned of fat and dissected from the corpus epididymis and the vas deferens. The cauda epididymis was minced and placed into 500 μL of HEPES-PSS buffer for 30 minutes in order to release spermatozoa into the buffer. Spermatozoa were centrifuged at 15000 rpm for 1 minute and re-suspended in 500 μL of HEPES-PSS buffer. Sperm were counted in a hemocytometer using 10 μL of the sperm suspension. A minimum of 100 sperms per chamber were counted to reduce statistical dispersion.

### Histology

Following fixation in 10% formalin, testis samples were dehydrated and embedded in paraffin. Five-micrometer transverse sections were stained using standard hematoxylin and eosin or Masson trichrome staining protocols (Sigma Aldrich, ON, Canada). Sections were viewed and imaged using an Olympus BX61 upright light microscope and Q imaging Retiga Exi camera with *InVivo* 3.2.0 software (Media Cybernetics). Computerized quantitative morphometric analysis was performed using Image-Pro Plus software (Media Cybernetics).

### Testicular morphometric analysis

Testicular volume was measured using the formula for a prolate (elongated) ellipsoid ($\frac{4}{3}\pi ab^{2}$) [30], where *“a”* is the major axis (polar diameter) and *“b”* is the minor axis (equatorial radius). The width and equatorial length of the testes were measured on testicular histological images using Image J1.45s, version 1.44p (NIH, Bethesda, MD). We found that testicular volume was significantly correlated with mouse size (R2 = 0.5, p = 0.02). Therefore, we divided testicular volume by the body weight of each mouse to yield volume per unit weight to normalize for mouse size.

Seminiferous tubule size was measured at a final magnification of 200X using Image-Pro Plus software (Media Cybernetics, Inc., Rockville, MD). To ensure comprehensive coverage of the testes, five rectangles of equal dimensions were randomly placed on the image. Within each of five grids placed on the histology image, 400 equidistant points were counted by manually tagging the cell type on which the point fell. A total of 2000 points were counted in each testis. If the point fell on white space within the seminiferous tubule, it was characterized as ‘intratubular space’, and if the point fell on white space outside the tubules, it was characterized as ‘interstitial space’. The lumen of the tubules was included as part of the spermatid count. Distinction was not made between a round or elongating spermatid. Germ cell count included both spermatogonia and spermatocytes. Sertoli cells, germ cells, and round and elongated spermatids in the seminiferous tubules were expressed as a percentage of the total cell population of the tubule. The cross section of the tubule in which cells were enumerated also included intracellular space. The density and distribution of Sertoli cells, germ cells, spermatids and Leydig cells within the testis were measured on a complete section at a final magnification of 200X using Image-Pro Plus software. Within each of the five rectangles, five tubules were randomly selected and the diameter of each axis (i.e. the lengths and widths) were measured. The five rectangles covered over 70% of the section and each rectangle had between 15–25 tubules within it so five tubules covered at least 20% of the tubules present. Tubules with a length-to-width ratio less than or equal to 1.5 were considered to be round or nearly round and were included in the analysis of seminiferous tubule size. At least 25 tubules were measured per testis (50 tubules per mouse) for both *Nf1*^*+/+*^ control and *Nf1*^*+/-*^ mice.

### Western blotting

Flash-frozen isolated testes from *Nf1*^*+/+*^and *Nf1*^+/-^ mice were homogenized in a pre-chilled stainless-steel mortar and pestle. The resulting tissue powder was mixed in 50 μL of ice-cold lysis buffer containing 50mM pyrophosphate, 50mM NaF, 50mM NaCl, 5mM EDTA, 5mM EGTA, 100μM Na_3_VO_4_, 10mM HEPES (pH 7.4), 0.1% Triton X-100, 10 μg/mL leupeptin, and 1mM phenylmethylsulfonyl fluoride. Extracted protein (40 μg) was fractionated by gel electrophoresis in 9% sodium dodecyl sulfate-polyacrylamide gels, transferred to nitrocellulose membranes, and then blocked for 1 hour with PBS containing 5% skim milk and 0.2% Tween-20. Following overnight incubation (at 4°C) with specific primary antibody, the membrane was incubated with secondary antibody for 1 hour at room temperature. Immunoblots were visualized with an enhanced chemiluminescence detection system following the protocol provided by the manufacturer (Pierce Biotechnology, Rockford, IL, USA).

Western blotting was performed with rabbit primary antibodies for p44/42 MAPK (ERK1/2), p44/42 MAPK phosphorylated at threonine 202 and tyrosine 204 (p-ERK1/2), or phosphatidylinositol 3-kinase (PI3K), along with anti-rabbit conjugated secondary antibody, all purchased from New England Biolabs (Whitby, Ontario, Canada).

### Statistical analysis

Data are reported as means ± SEM from at least five mice. Differences between *Nf1*^*+/+*^ and *Nf1*^*+/-*^ groups were analyzed by 2-tailed Student’s t tests and were graphed using GraphPad 5 Prism software (San Diego, Calif., USA). Statistical significance was defined as p ≤ 0.05.

## Results

### Effects of Nf1 mutation on mouse litter size and sperm count

To assess the fertility of *Nf1*^+/-^ male mice, we determined the number of pups produced when *Nf1*^+/-^ males were crossed to *Nf1*^*+/+*^ control females, and *Nf1*^*+/+*^ control males were crossed to *Nf1*^+/-^ females. As shown in Table 1, the average number of pups per litter from *Nf1*^+/-^ males was significantly reduced compared to that of *Nf1*^*+/+*^ control male littermates (p = 0.007); however, the amount of time between litters was not significantly different (p = 0.75). We saw no evidence of post-natal death or other indications that *Nf1*^*+/-*^ males were more aggressive with pups than *Nf1*^*+/+*^ control males. Epididymal sperm counts in *Nf1*^+/-^ mice were significantly higher than those of *Nf1*^*+/+*^ control mice (p = 0.02) **(Table 1).**

**Table 1:** Testicular Functional Parameters in *Nf1*^*+/+*^ and *Nf1*^*+/-*^ mice. Values listed in each column are mean ± SEM. Bold numbers indicate p≤ 0.05 and are considered to be statistically significant.

Testicular Functional Parameter	Nf1^+/+^ male x Nf1^+/-^ female	Nf1^+/-^ male x Nf1^+/+^ female	p-value
**Average Litter Size**	8.67 ± 0.76	4.00 ± 1.2	0.007
**Days between Litters**	33.4 ± 4.7	31.2 ± 4.9	0.75
**Sperm Count (x10**^**6**^ **spermatozoa/ml)**	8.8 ± 5.5	15 ± 2.3	0.02

### Histological changes in seminiferous tubules

**Fig 1:**
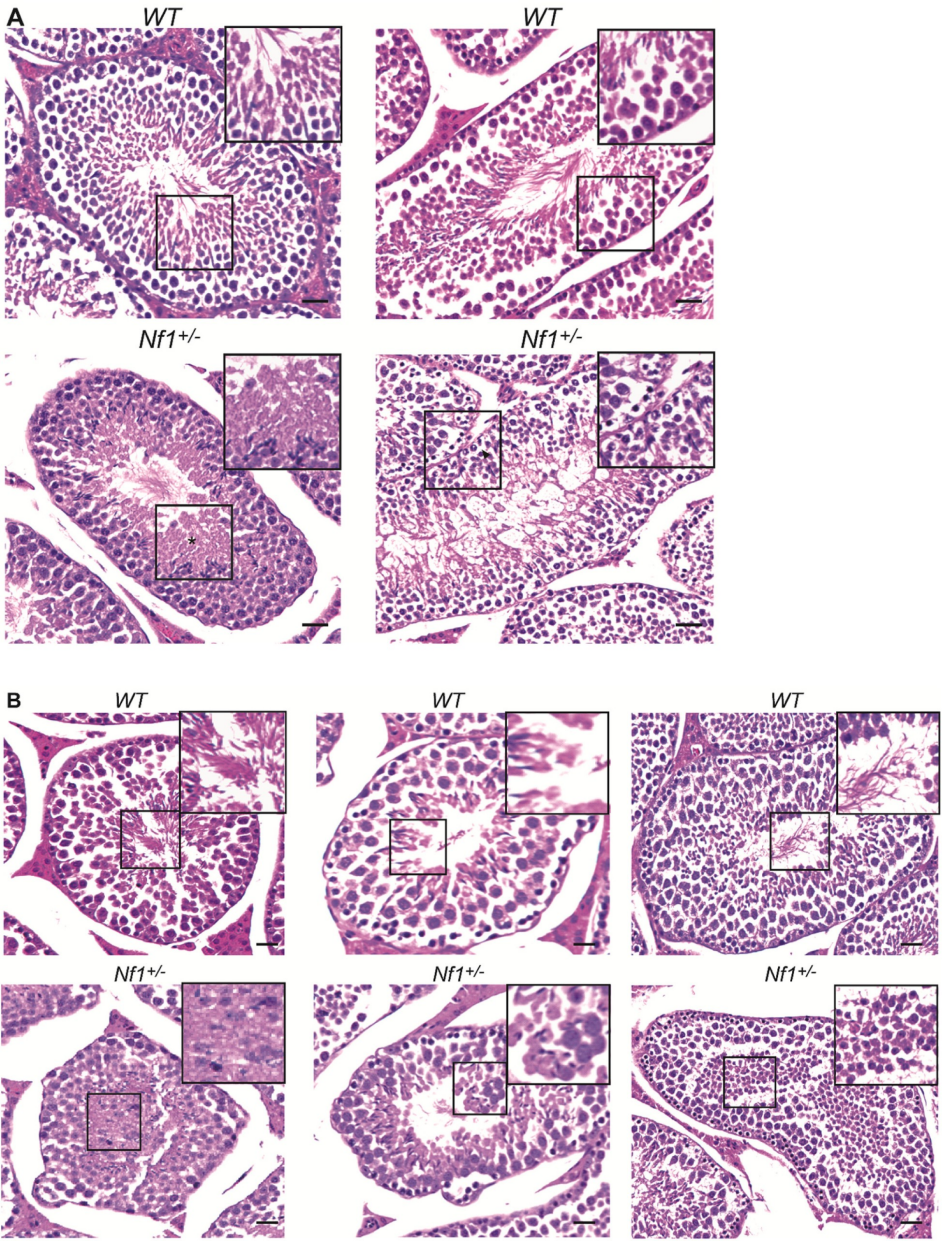
Seminiferous tubule degeneration in *Nf1*^+/-^ mice. (A) Photomicrographs from *Nf1*^*+/+*^ (upper panels) and *Nf1*^+/-^ (lower panels) seminiferous tubules taken at 200X showing mild to moderate degeneration of seminiferous tubules in *Nf1*^+/-^ mice (detachment of germ cells from the basal epithelium, vacuolation of the seminiferous tubule and presence of atypical residual bodies. (B) Photomicrographs from *Nf1*^*+/+*^ (upper panels) and *Nf1*^+/-^ (lower panels) testes taken at 200X showing moderate alterations in spermatogenesis in *Nf1*^+/-^ mice (exfoliation of immature round spermatids and germ cells into the lumen of seminiferous tubules). (Scale bar; 50μm).

To determine whether these changes in fertility were associated with morphological changes in the testes, we performed histological examination of the testis in *Nf1*^+/-^ males compared to *Nf1*^*+/+*^ control males. We found varying amounts of mild to moderate degeneration of seminiferous tubules in all *Nf1*^+/-^ males. Mild alterations include detachment of the germinal epithelium from the basal lamina, vacuolation, and residual bodies **(Fig 1A)**. Moderate alterations include disorganization of the seminiferous epithelium, with exfoliation of germ cells and immature spermatids into the lumen **(Fig 1B)**, suggesting that spermiation may be affected.

### Effects of Nf1 mutation on spermatid maturation

**Fig 2:**
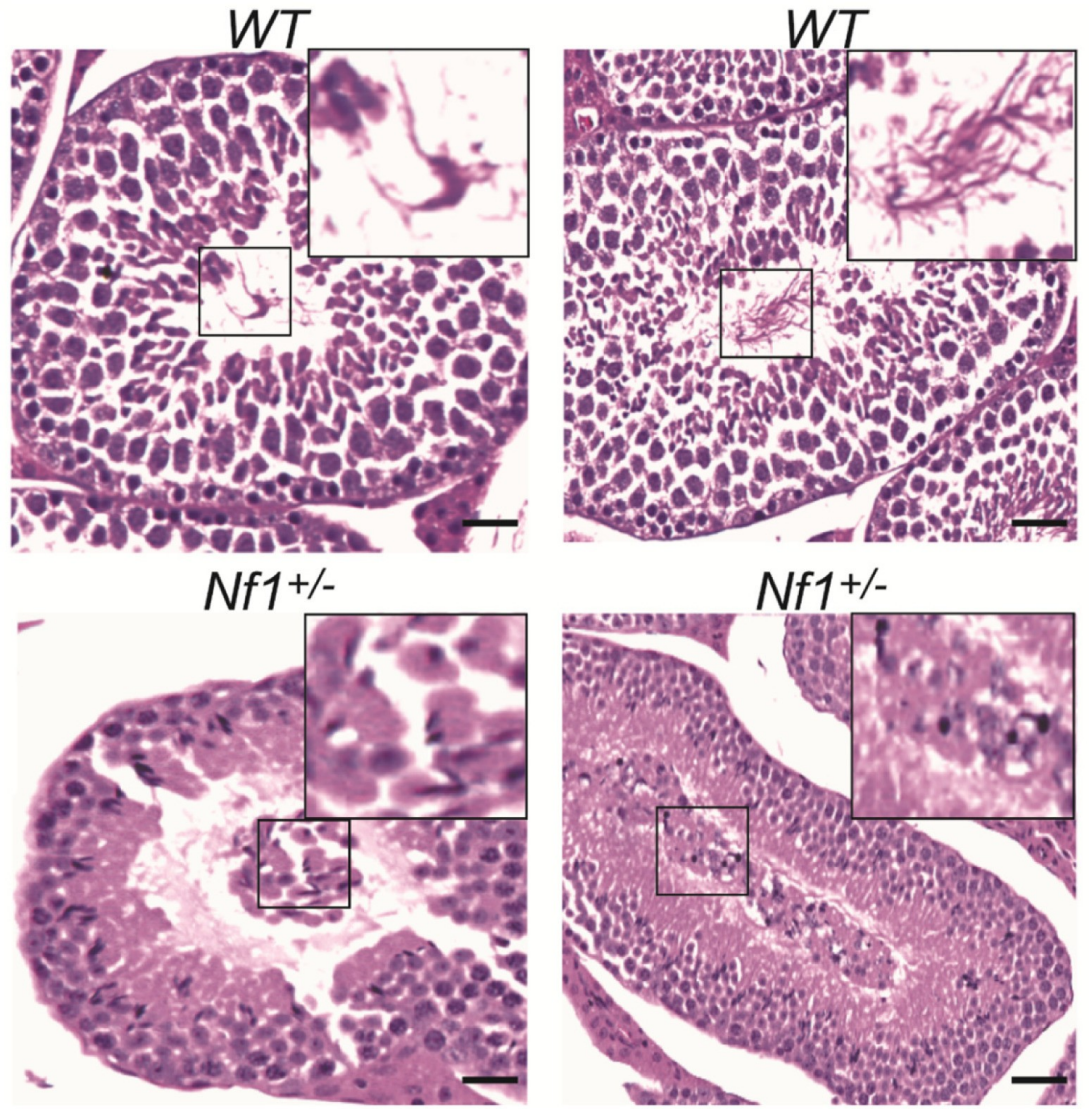
Alterations in spermiation in *Nf1*^+/-^ mice. Photomicrographs from *Nf1*^+/-^ testes taken at 200X showing abnormal spermatid morphology (*) (enlarged heads and presence of residual bodies) associated with alterations in spermiation in *Nf1*^+/-^ mice. (Scale bar; 50μm).

Further suggesting an important role for neurofibromin during spermiation, we also observed abnormal spermatids with enlarged heads and atypical residual bodies in *Nf1*^+/-^ mice, which appear to be associated with the release of immature spermatids into the lumen (**Fig 2**).

Progressive differentiation and migration of spermatocytes is required for the production of mature spermatozoa. To further examine this process, we quantified the number of Leydig cells, Sertoli cells, spermatocytes, and spermatids in the seminiferous epithelium of *Nf1*^+/-^ and *Nf1*^*+/+*^ control mice **(Table 2).**

**Table 2:** Testicular structural parameters in *Nf1*^*+/+*^ control and *Nf1*^*+/-*^ mice. Values listed in each column are mean ± SEM. Values represent the average per field counted, except for testicular volume and seminiferous tubule area, which are per testis or per tubule, respectively. Bold numbers indicate p≤ 0.05.

Testicular Structural Parameters	Nf1^+/+^ n = 5	Nf1^+^ n = 6	p-value
**Number of Leydig cells**	28.2 ± 0.71	24.2 ± 1.2	0.02
**Amount of Interstitial Space**	68.7 ± 6.7	74.6 ± 3.2	0.43
**Number of Sertoli cells**	0.027 ± 0.004	0.025 ± 0.005	0.75
**Number of Germ cells**	0.32 ± 0.01	0.34 ± 0.008	0.13
**Number of Round and Elongated Spermatids**	0.51 ± 0.02	0.56 ± 0.008	0.03
**Testicular Volume**	2.73 ± 0.09	3.11 ± 0.14	0.05
**Seminiferous Tubule Area**	52720 ± 3208	47990 ± 2138	0.25

Leydig cells secrete testosterone and cytokines required for spermatogenesis. The number of Leydig cells present in *Nf1*^+/-^ mice was significantly lower than in *Nf1*^*+/+*^ control littermates (p = 0.02) **(Table 2)**. This reduction did not result in a significant increase in the interstitial space in *Nf1*^+/-^ mice and was not associated with any difference in the number of Sertoli cells. The total number of germ cells also did not differ between *Nf1*^+/-^ and *Nf1*^*+/+*^ control mice, although the number of immature (round or elongated) spermatids was significantly increased in *Nf1*^+/-^ mice compared to *Nf1*^*+/+*^ control (p = 0.03) **(Table 2)**. Testicular volume was also significantly increased among *Nf1*^+/-^ mice (p = 0.05) **(Table 2)**. This difference does not appear to be a result of alterations in the size of the seminiferous tubules, as we did not observe a significant difference in the seminiferous tubule area between *Nf1*^+/-^ and *Nf1*^*+/+*^ control mice **(Table 2)**.

### Effects of the Nf1 mutation on signaling pathways within seminiferous epithelium

**Fig 3:**
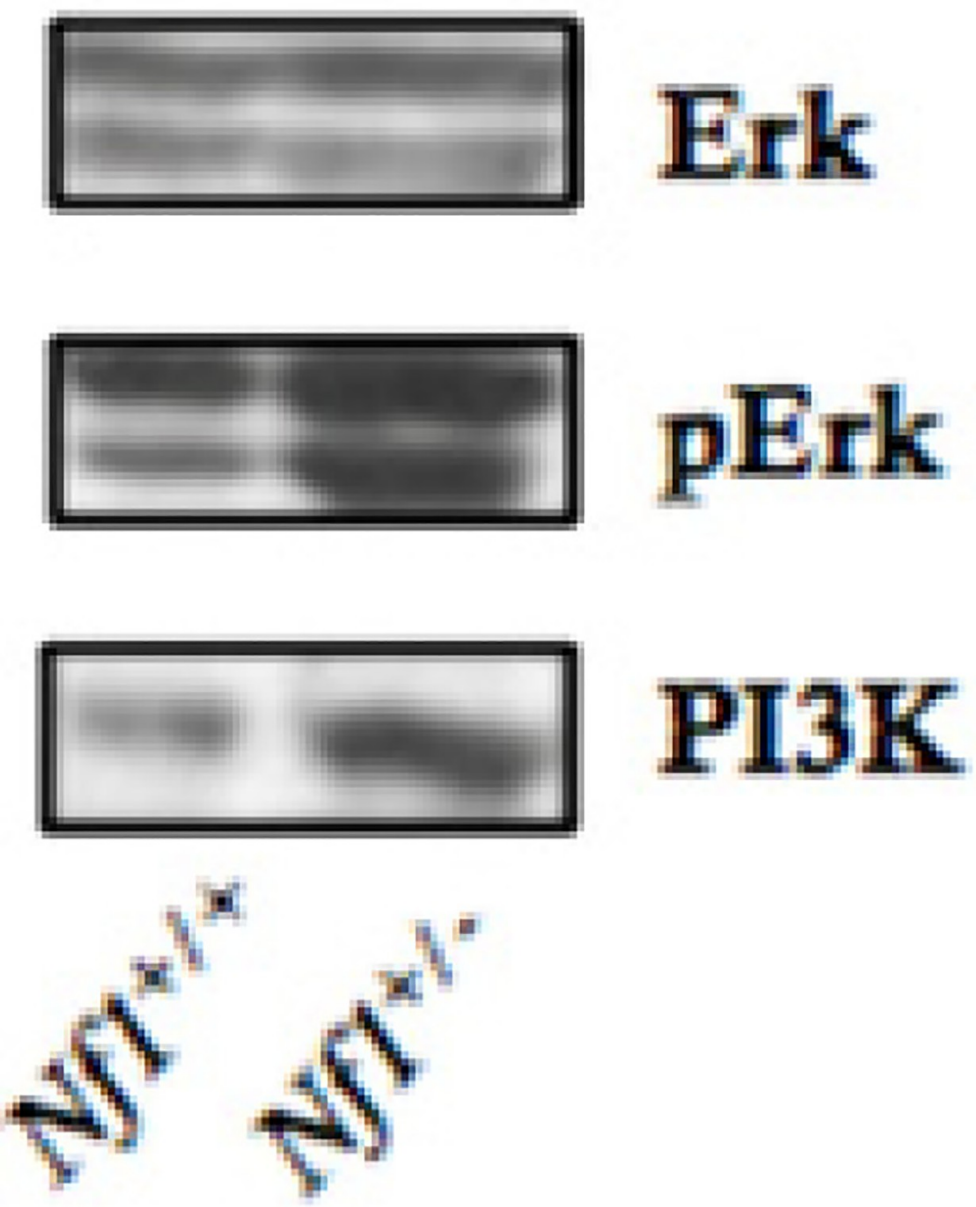
Up-regulation of PI3K expression and ERK phosphorylation in *Nf1*^+/-^ mice. Western blot analysis of protein expression showing up-regulation in PI3K and phosphorylated ERK (pERK) with similar ERK expression in *Nf1*^+/-^ testes compared to *Nf1*^*+/+*^ testes.

As loss of neurofibromin may cause hyperactivation of the RAS/MAPK/ERK pathway [16, 19, 28], which is involved in regulation of seminiferous epithelium [20–23, 31]. We examined the effects of neurofibromin deficiency on expression and activation of the signaling components of the phosphoinositide 3-kinase (PI3K) and ERK pathway in the testes, and showed that *Nf1* mutation resulted in increased phosphorylation and activation of ERK, while having no effect on total ERK protein expression (**Fig 3).** In addition, we observed a significant increase in total PI3K expression in *Nf1*^+/-^ mice when compared to *Nf1*^*+/+*^ control mice **(Fig 3)**.

## Discussion

Despite reproductive fitness being reduced in individuals with NF1 [2–4, 6], gonadal function has not been well characterized. Delayed puberty [9] and early menopause [11] suggest that reproductive function may be altered in people with NF1.

The present work is the first time that testicular morphology and function have been examined in *Nf1*^*+/-*^ mice. Interestingly, we observed a 50% reduction in the average litter size from *Nf1*^+/-^ males crossed to *Nf1*^*+/+*^ control females when compared to *Nf1*^*+/+*^ control males crossed to *Nf1*^+/-^ females. This suggests fertility may be reduced in *Nf1*^+/-^ males, but we cannot rule out the possibility that the effect observed is due to an increase in the fertility of *Nf1*^+/-^ females when paired to *Nf1*^*+/+*^ control males. However, as described below, the reduced litter size in these crosses was associated with changes in spermatogenesis and sperm quality in *Nf1*^+/-^ males, supporting a role for *Nf1* in male fertility.

Changes in spermatogenesis and/or sperm quality may be associated with alterations in the seminiferous epithelium, which are known to affect fertility rates [32, 33]. In models of testicular toxicity, the disorganized seminiferous epithelium appears to result from alterations in Sertoli-spermatocyte and Sertoli-spermatid junctions [14, 17, 34, 35]. Spermatogenesis depends on the maintenance and restructuring of cellular junctions between Sertoli cells, spermatocytes, and spermatids [36]. In the testis, cellular junctions regulate the permeability of amino acids, hormones, and growth factors, and create an immune barrier essential for prevention of antigen responses [33].

Coexisting tight junctions and a special adherens junction, known as the basal ectoplasmic specialization, form the blood-testis barrier [36]. Spermatocyte differentiation depends on the migration of immature spermatocytes across the blood-testis barrier and progressive migration toward the seminiferous tubule lumen. During this process, Sertoli-Sertoli, Sertoli-spermatocyte, and Sertoli-spermatid cell junctions are rapidly remodeled to enable differentiation and migration of spermatocytes and to maintain the integrity of the blood-testis barrier [21]. Alterations in Sertoli-spermatocyte junctions cause exfoliation of germ cells [14], while exfoliation of immature spermatids into the seminiferous tubule lumen suggests that Sertoli-spermatid junctions are affected as round immature spermatids would normally associate with Sertoli cells via the ectoplasmic specialization [37]. We observed exfoliation of spermatocytes and round and elongating spermatids into the lumen, suggesting that neurofibromin haploinsufficiency may affect junctional dynamics in the testis.

Further contributing to the exfoliation of immature round and elongated spermatids in *Nf1*^+/-^ mice, we also found that the number of Leydig cells was also reduced in *Nf1*^*+/-*^ mice. Leydig cells produce testosterone which reinforces the tight junctions in the blood-testis barrier [15, 21, 38] and regulates ES assembly and disassembly [14, 16, 37]. Testosterone withdrawal promotes stage specific-detachment of round spermatids from the rat seminiferous epithelium through an ERK-dependent mechanism [39]. *Nf1*^*+/-*^ mice also demonstrated enlarged sperm heads and atypical residual bodies, which further supports a role for neurofibromin in junctional dynamics. Alterations in spermatid morphology usually result from disassembly of tubulobulbar complexes, a specialized junction between Sertoli cells and elongated spermatids [40].

ERK phosphorylation is associated with the basal ectoplasmic specialization, a Sertoli-spermatocyte adherens junction that regulates spermatogenesis, and the apical ectoplasmic specialization, a Sertoli-spermatid adherens junction involved in spermiation [15, 32]. Phosphorylation of ERK results in decreased levels of the adhesion protein complexes, cadherin/catenin and nectin/afadin, resulting in loss of ectoplasmic specialization adhesion [14, 25]. Increased phosphorylation of ERK because of neurofibromin haploinsufficiency is, therefore, likely to affect proliferation of immature spermatogonia and affect ES adhesion. In support of this hypothesis, we found that in *Nf1*^*+/-*^ mice, there was an abnormal release of immature round spermatids from the seminiferous epithelium and an increase in spermatids were associated with a significant increase in ERK phosphorylation without affecting total ERK protein expression.

Though alterations in spermiation may result in lower sperm counts, we found that caudal epididymal sperm counts in *Nf1*^*+/-*^ mice were significantly increased. It is conceivable that the hyperactivation of Ras affects survival of spermatids and contributes to a higher sperm count yet lower quality sperm. Activation of ERK is associated with reduction in sperm quality [21] and alterations in sperm quality are known to reduce the number of offspring produced [41, 42]. The presence of abnormal spermatid morphology such as atypical residual bodies in the spermatids of *Nf1*^+/-^ mice further supports the hypothesis that *Nf1*^+/-^ mice produce lower quality sperm. Reduced reproductive fitness with an increased sperm count is not frequently observed, but has been demonstrated in progesterone receptor knockout mice (PRKO) with a similar phenotype to *Nf1*^*+/-*^ male mice [43, 44]. Similar to *Nf1*^*+/-*^ mice, PRKO mice demonstrate delayed spermatogenesis [43] with alterations in Leydig cells [44]. While sperm quality was not directly assessed in this model, in humans reduced progesterone receptor expression is known to affect sperm quality [45].

Neurofibromin is a negative regulator of Ras, and neurofibromin haploinsufficiency leads to activation of the Ras pathway [1, 19, 28]. Activation of Ras is known to trigger phosphorylation of ERK, which promotes spermatogonial stem cell proliferation in mice [26, 27] and is involved in differentiation of spermatogonia through regulation of adherens junctions. We speculate that the hyperactivation of ERK may also contribute to abnormal loss of ectoplasmic specialization adhesion during spermiation. Further highlighting the importance of ERK in spermatogenesis, MAP3K, the human ortholog to ERK1, is highly expressed in Leydig cells and moderately expressed throughout the seminiferous tubules in adult males (see https://www.proteinatlas.org/ENSG00000102882-MAPK3/tissue/testis#img).

Together, the results from this study demonstrate that *Nf1*^*+/-*^ mice have alterations in the seminiferous epithelium, and suggests that neurofibromin is involved in proliferation of the seminiferous epithelium, as well as regulation of the junctions vital to spermatogenesis. The precise mechanism of the alterations observed here is unclear, but *Nf1*^*+/-*^ mice do appear to demonstrate defects in spermatogenesis. Thus, the observed alterations in the seminiferous epithelium of *Nf1*^*+/-*^ mice may be related to reduction in fertility, but further investigation is needed to establish the causal relationship.

At this point, we do not know if similar alterations are present in patients with NF1, testosterone levels and spermatogenesis are rarely examined unless a symptomatic testicular abnormality is also present [46–48]. However, fertility is reduced [3], puberty is delayed, and osteoporosis appears to occur more frequently than expected in men with NF1 [11] suggesting defects in gonadal function or endocrine regulation.

Although there are discrepancies in spermatogenesis between human and mice, in light of the facts and findings detailed in this study, investigation of testicular function in patients with NF1 may be warranted. These studies should explore sperm counts, testosterone levels, and morphometric measurements of the testes as well as testicular pathology. With this knowledge, patients can be counselled to make decisions regarding their reproductive potential. Examination of ovarian structure and function in female *Nf1*^+/-^ mice is also worth pursuing, as we are unaware of any studies that have examined it.
